# Data on SiC-based bundle lifetime variability: The insufficiency of external phenomena affecting the flaw size

**DOI:** 10.1016/j.dib.2021.106757

**Published:** 2021-01-15

**Authors:** S. Mazerat, R. Pailler

**Affiliations:** Univ. Bordeaux, CNRS, CEA, SAFRAN CERAMICS, LCTS, UMR 5801, F-33600 Pessac, France

**Keywords:** Slow crack growth, Delayed failure, Bundle model, SiC-based fiber, Nicalon, Tyranno

## Abstract

A broad variability characterizes the lifetime of SiC-based bundles under static fatigue conditions at intermediate temperature and ambient air, challenging the accuracy of its prediction. The same is true, in a lower extend, with tensile properties, in apparent discrepancy with the bundle theory based on weakest link theory. The data presented here focus on lifetime scattering, evaluated on different fiber types (6 in total, Nicalon® or Tyranno®). It is hosted at http://dx.doi.org/10.17632/96xg3wmppf.1 and related to the research article “Static fatigue of SiC-based multifilament tows at intermediate temperature: the time to failure variability” (Mazerat et al., 2020) [1]. The insufficiency of classically invoked external and discrete bias (fiber sticking phenomenon for instance) was compared to a devoted Monte Carlo algorithm, attributing to each filament a strength (random) and a stress (homogeneous). Introduction of a stress inconsistency from tow to tow, experimentally observed through section variability, was revealed to overpass such biasing approach. This article can be referred to for the interpretation or prediction of CMC lifetime to guaranty long term performances over the broad offered application field.

## Specifications Table

**Table:** 

Subject	Material Science
Specific subject area	Delayed failure of SiC/SiC ceramic matrix composite
Type of data	Figure Table
How data were acquired	Static fatigue data were acquired suspending a dead weight to a bundle, placed in a resistive furnace opened to ambient atmosphere, and measuring the time before its failure.
Data format	Raw and analysed
Parameters for data collection	Bundles were repeatedly tested under static fatigue conditions in a 500–1500 MPa stress range at intermediate temperature (500–900 °C).
Description of data collection	Monte Carlo simulated lifetime scattering, based on bundle model with strength or stress bias, was compared to experimental dataset.
Data source location	Univ. Bordeaux, CNRS, CEA, SAFRAN CERAMICS, LCTS, UMR 5801, F-33600 Pessac, France
Data accessibility	With the article and to http://dx.doi.org/10.17632/96xg3wmppf.1
Related research	S. Mazerat, R. Pailler, Static fatigue of SiC-based multifilament tows at intermediate temperature: the time to failure variability, Int. J. Fatigue. In Press https://doi.org/10.1016/j.ijfatigue.2020.106072

## Value of the Data

• This dataset is valued because it gives some insight onto the sources of SiC-bundle lifetime dispersion and limitation of some approaches invoked to interpret it.
• The data can be used for comparative and comprehensive works on static fatigue behavior of SiC filaments or tows.
• The dataset can assist the understanding of bundle strength and lifetime variability sources. It may also argue the selection of a reinforcement type for a given application (design on purpose).
• These data bring new insight onto the interpretation of such variability, introducing the uncertainty on applied stress in a Monte Carlo based simulation model.

## 1 Data Description

**Fig. 1:**
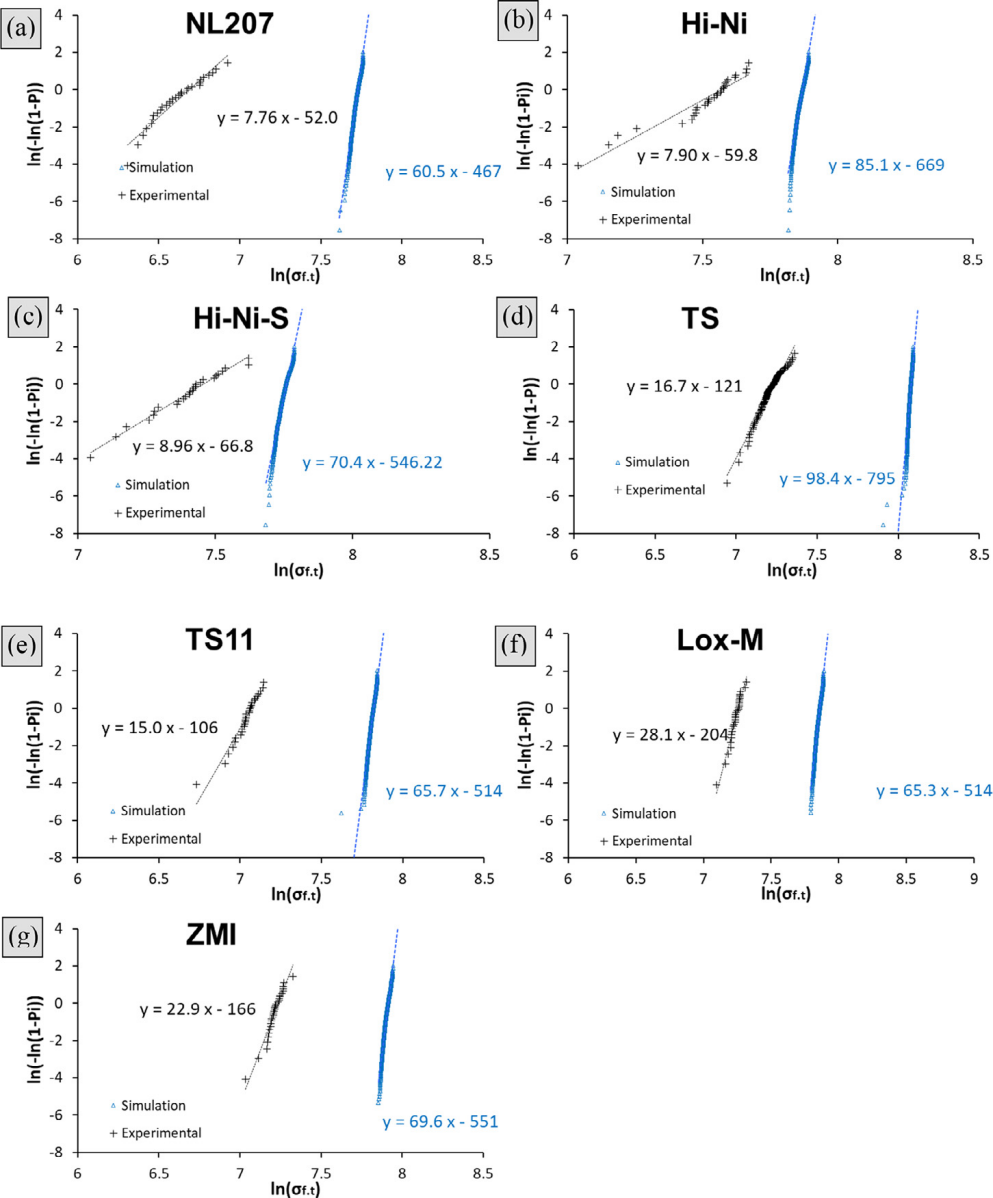
Weibull plots for experimental and simulated bundle tensile strength on (a). NL207 (b). Hi-Ni (c). Hi-Ni-S (d). TS (e). TS11 (f). Lox-M and (g). ZMI. Simulation was based on bundle model algorithm, with a failure dictated by the filament strength of rank *α_c_* (Eq. (5)).

The dataset described herein analyzes the scatter experienced by extensive tensile or static fatigue testing of SiC-based bundles. A total of 6 fiber types were investigated: Nicalon® NL207, Hi-Nicalon®, Hi-Nicalon® Type S, Tyranno® Grade S, Tyranno® Lox-M and Tyranno® ZMI. It was deemed necessary to build supplementary figures and share the raw data to highlight the relevance of observations done in Ref. [1]. Raw data and the algorithm are available in Mendeley data repository under the following identifier DOI:10.17632/96xg3wmppf.2. As a preliminary study, bundle tensile behavior was investigated. The ruin under monotonous strain rate was therefore considered to be governed by a critical filament of rank *α_c_* (cf. method section, Eq. (5)). The stochastic character of strength ascribed to each of the *N_0_* filaments was repeated 1000 times to estimate the distribution parameter for virtual bundle strength. The latter were finally compared to experimental datasets from [2] (Fig. 1). A drastic underestimation of the tow strength and its dispersion can be noticed. The Weibull modulus calculated on virtual bundle exceeded (2 to 10 times larger) the actual ones as shown in Table 2.

**Fig. 2:**
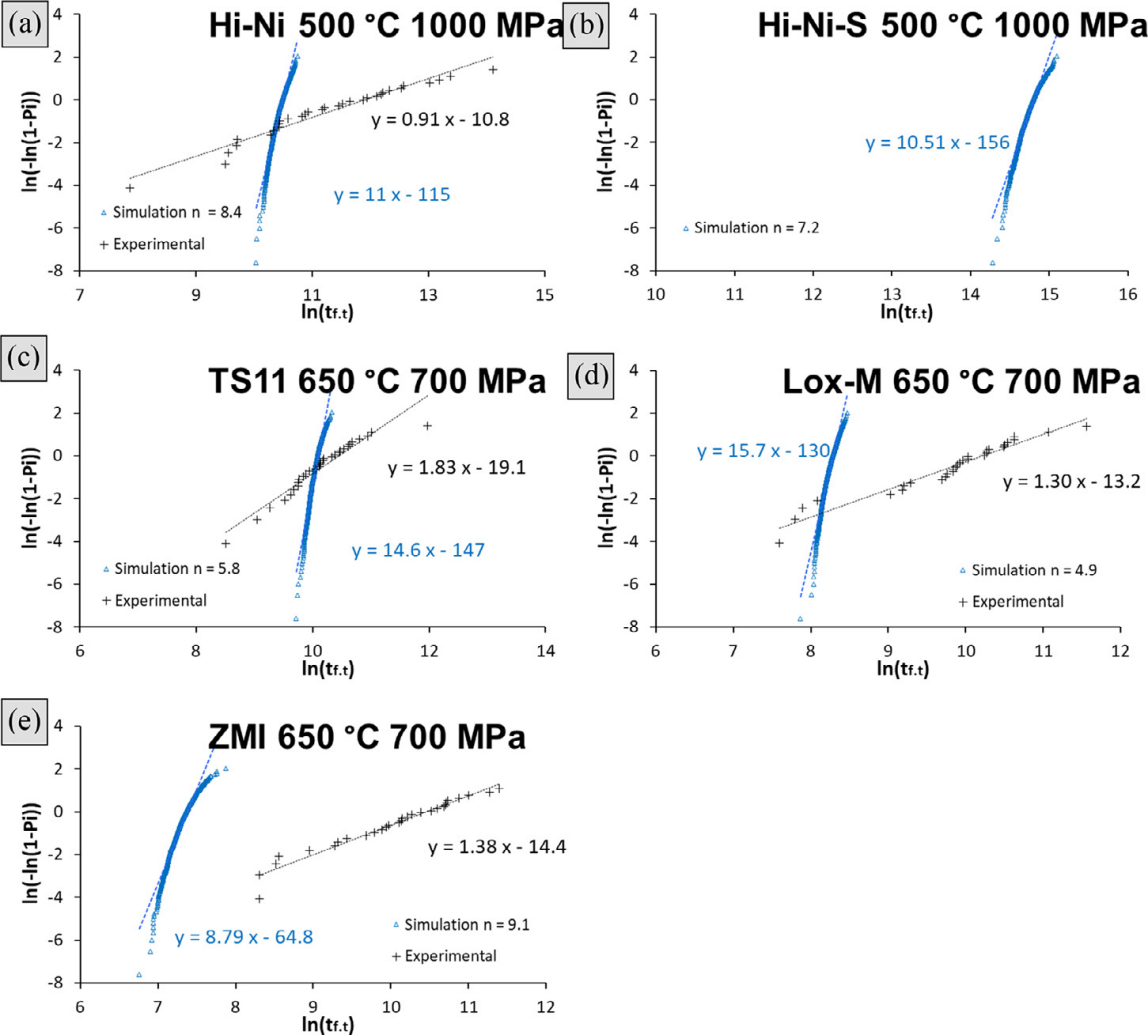
Weibull plots comparing experimental and simulated lifetime dispersions for (a). Hi-Ni (b). Hi-Ni-S both at 500 °C and 1000 MPa (c). TS11 (d). Lox-M (e). ZMI at 650 °C and *σ_app.t(γ=0%)_* = 700 MPa. Simulation was based on bundle model (same stress applied to each fiber), with a failure dictated by the filament of lifetime or strength rank α_t_ (Eq. (9)).

**Fig. 3:**
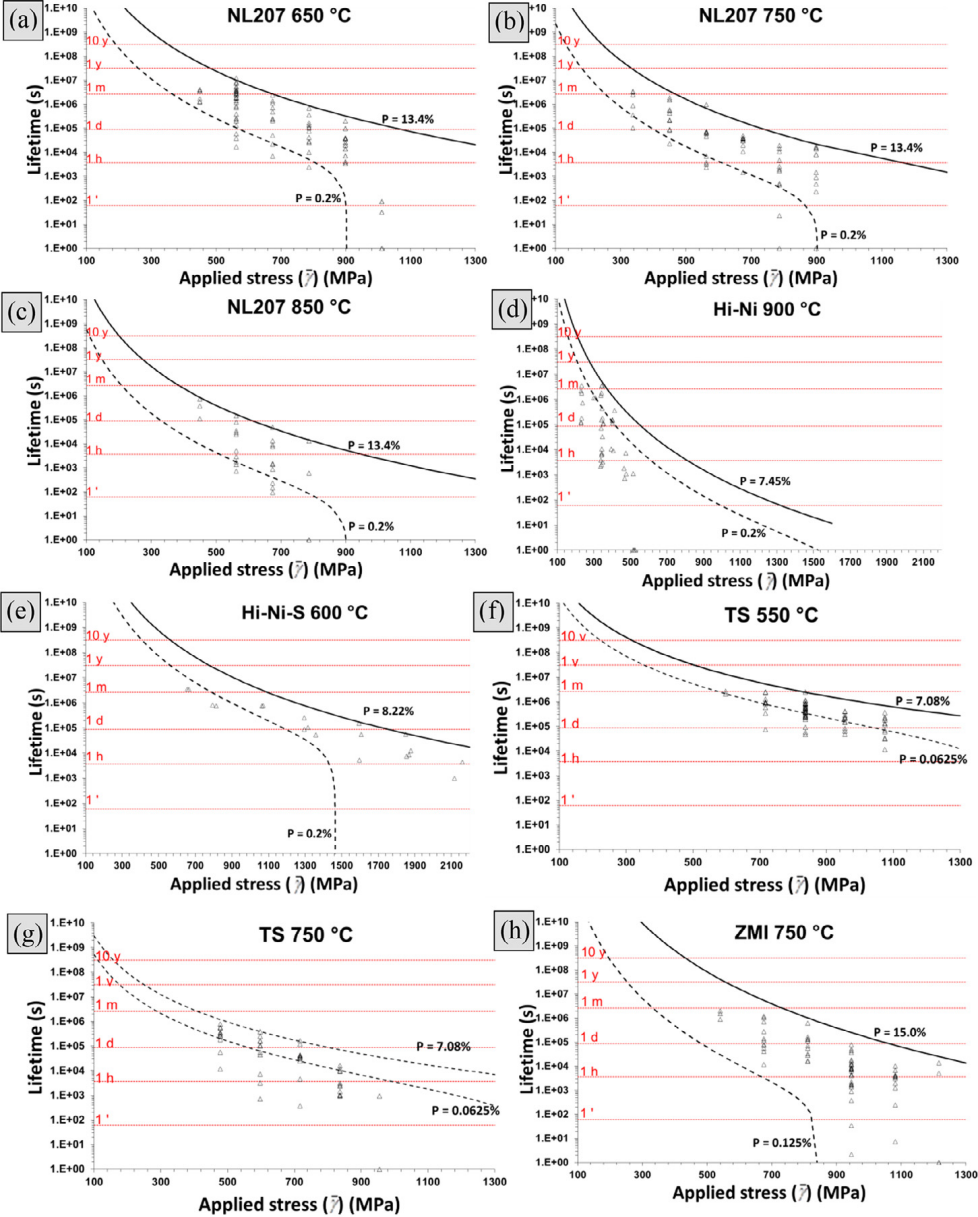
Endurance diagrams showing the prediction range taking α_t_ (upper limit) or the weakest filament (1/*N_t_*) into consideration for NL207 at (a). 650 °C (b). 750 °C (c). 850 °C, (d). Hi-Ni at 900 °C, (e). Hi-Ni-S at 600 °C, TS at (f). 550 °C (g). 750 °C and (h). ZMI at 750 °C. *A_1_* coefficients (respectively 38, 580, 2300, 5900, 0.18, 6.6, 220, 520 × 10^−12^ m^1-n/2^ MPa^−^*^n^* s^−1^) were taken so the upper limit fits experimental data.

A similar work was then performed under static fatigue conditions, with an invariant γ value and taking only the filament strength variability into account. Here, the bundle ruin was considered to happen when a critical fraction *α_t_* Eq. (16), gathered in Table 3) of fibers had failed by subcritical crack growth. An example of this simulation tool applied to NL207 type (‘Monte Carlo simulation NL207.xlsx’) are given in the supplementary file. A Set of 50 bundles are randomly generated, each one having a unique architecture. The lifetime variability extracted for this algorithm was drastically underestimating (Weibull moduli >5 times larger) tests results as shown on Weibull diagrams (Fig.  2) and as mentioned in [1]. The computed and experimental raw data used to build these diagrams and assess the virtual distribution can be found in the supplementary file ‘Variability comparison.xlsx’ for all fiber types. The first attempt to interpret this scatter underestimation is to consider a fiber sticking-induced bias on α_t_ fraction as described in [3]. Therefore, the upper lifetime limit was fitted by the prediction model (Eqs. (16), ((18) with parameters given in Table 3) and lower bonds considered piloted by the weakest filament of the tow (bonded to a critical amount of fibers, *α_t_* = 1/*N_t_*). This approach could however not encompass the data points for all fiber types as shown on endurance diagrams in Fig. 3: on Hi-Nicalon and Grade S types a drastic underestimation is observed. NL207, Hi-Nicalon type S and ZMI however show better consistency. This is to be linked with Weibull statistical parameters for the fiber strength (*m_f_* >6 on TS and Hi-Ni, Table 1) [1]. Also, no particular tendency could be noticed when trial temperature was increased as shown on NL207 comparing the results at 650, 750 and 850 °C (Fig. 3a–c).

**Table 1:** Statistical parameters describing the distributions of unloaded section fraction (*γ*) and filament strength.

	*m_f_*	σ_1.f_ (MPa)	$\bar{\gamma }$ (%)	*m*_γ_	*γ_0_* (%)
NL207	5.05	3090	10 ± 10	2.44	28.9*
Hi-Ni	9.84	3295	14 ± 12	2.00	27.3*
Hi-Ni-S	8.42	3062	25 ± 16	1.36	29.0
TS	8.17	3550	16 ± 11.2	1.48	22.8
TS11	4.79	3034	10 ± 8.5	1.33	11.8
Lox-M	4.63	3314	25 ± 8.1	1.38	15.6*
ZMI	4.81	3372	14 ± 8.5	2.29	23.6*

⁎ indicates the data that were offset as given in [2]. These values are duplicated from the related research article [1].

**Table 2:** Experimental and simulated Weibull modulus for bundle tensile strength.

		Experimental		Simulation	
	α_c_ (%)	m_t_	σ_0.t_ (MPa)	m_t_	σ_0.t_ (MPa)
NL207	18.0	7.8	810	61	2270
Hi-Ni	9.7	7.9	1930	85	2620
Hi-Ni-S	11.2	9.0	1730	70	2340
TS	11.5	17	1380	98	3225
TS11	18.8	15	1180	66	2490
Lox-M	19.4	28	1420	65	2590
ZMI	18.8	23	1390	70	2710

**Table 3:** Parameters used to construct the Fig. 3 strength of fiber with rank *α_t_* or 1/*Nt* and *A_1_* coefficient empirically adjusted to fit upper lifetime limit.

	Upper lifetime			Lower lifetime
	*α_t_* (%)	*σ_f.f(αt)_* (MPa	*A*_1_ (m^1-^*^n^*^/2^ MPa^−^*^n^* s^−1^)	*σ_f.f(αt=1/N0)_* (MPa)
NL207 650 °C	13.4	2100	3.8 × 10^−11^	900
NL207 750 °C	13.4	2100	5.8 × 10^−10^	900
NL207 850 °C	13.4	2100	2.3 × 10^−9^	900
Hi-Ni 900 °C	7.4	2540	5.9 × 10_–9_	1750
Hi-Ni-S 600 °C	8.2	2290	1.8 × 10^−13^	1460
TS 550 °C	7.1	2580	6.6 × 10^−12^	1440
TS 750 °C	7.1	2580	2.2 × 10^−10^	1440
ZMI 750 °C	15.0	2310	5.2 × 10^−10^	840

**Fig. 4:**
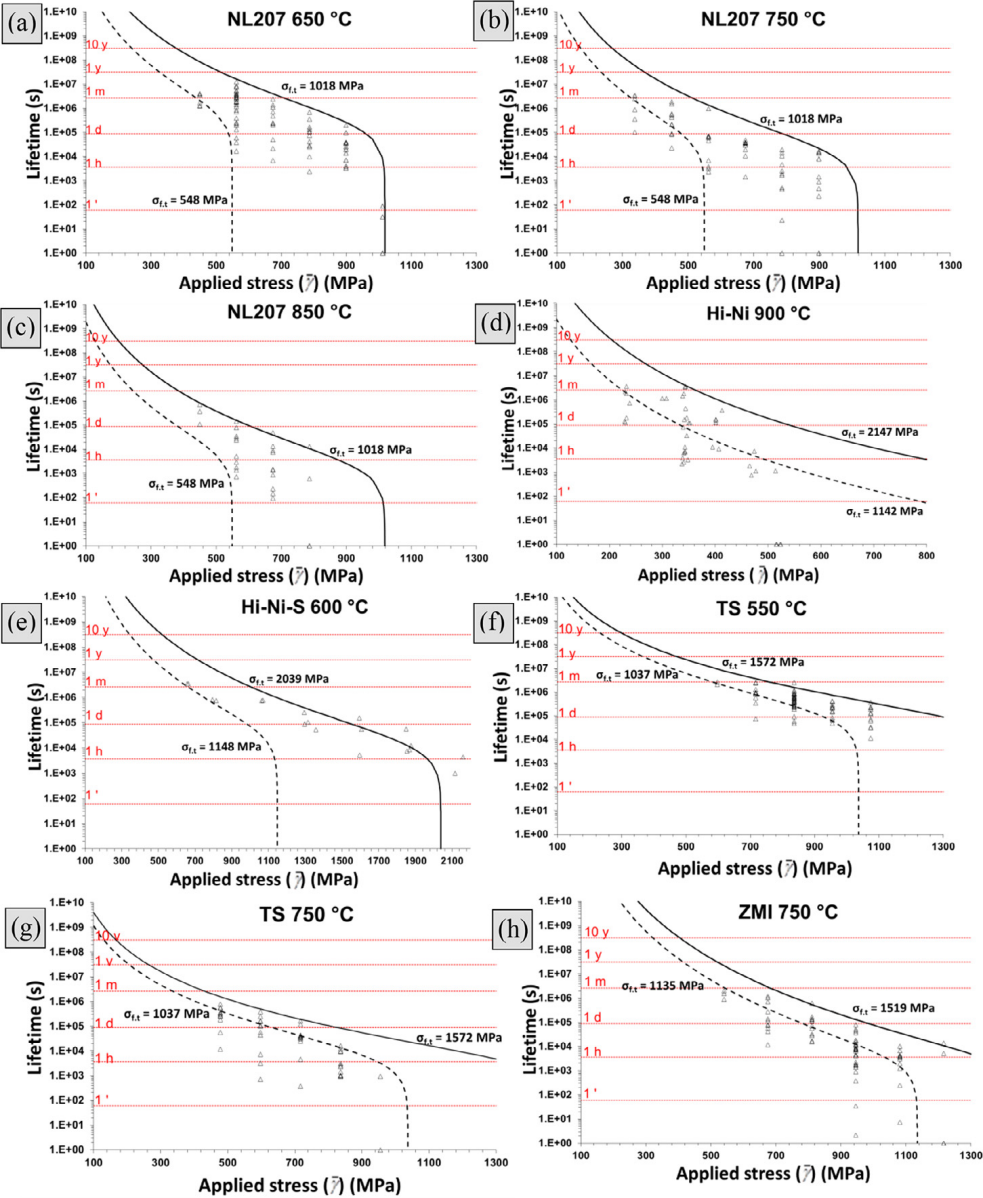
Endurance diagrams for NL207 at (a). 650 °C (b). 750 °C (c). 850 °C, (d). Hi-Hi at 900 °C, (e). Hi-Ni-S at 600 °C, TS at (f).550 °C (g). 750 °C and (h). ZMI at 750 °C showing the prediction range using tow strength range. *A*_1_ parameters (respectively 0.49, 6.0, 50, 2500, 0.18, 2.0, 37, 50 × 10^−12^ m^1-^*^n^*^/2^ MPa^−^*^n^* s^−1^) were taken so upper limit fits higher lifetime.

Introduction of bundle tensile strength in place of the critical filament strength in Eq. (13), based on the over-estimation of this latter as shown in Fig. 1 was investigated. Values for higher and lower tow strength were extracted from a previous data article [2] and summarized in Table 4. Here, again, results on Grade S and Hi-Nicalon underestimate the experience scattering, accompanied by ZMI (Fig. 4). In contrast, NL207 and Hi-Nicalon S are more consistent. With this approach however, the scatter would be expected to decrease at lower applied stress, which is not evidenced on test results (Fig. 4b).

**Table 4:** Summary of upper and lower bundle strengths used to construct the Fig. 4. *A_1_* was empirically adjusted so the predicted lifetime with *σ_f.t_*_max_ fitted the upper data point range.

	Upper lifetime		Lower lifetime
	σ_f.t max_ (MPa)	A_1_ (m^1-^*^n^*^/2^ MPa^−^*^n^* s^−1^)	σ_f.t min_ (MPa)
NL207 650 °C	1020	4.9 × 10^−13^	550
NL207 750 °C	1020	6.0 × 10^−12^	550
NL207 850 °C	1020	5.0 × 10^−11^	550
Hi-Ni 900 °C	2150	2.5 × 10^−9^	1140
Hi-Ni-S 600 °C	2040	1.8 × 10^−13^	1150
TS 550 °C	1570	2.0 × 10^−12^	1040
TS 750 °C	1570	3.7 × 10^−11^	1040
ZMI 750 °C	2310	5.2 × 10^−10^	840

**Fig. 5:**
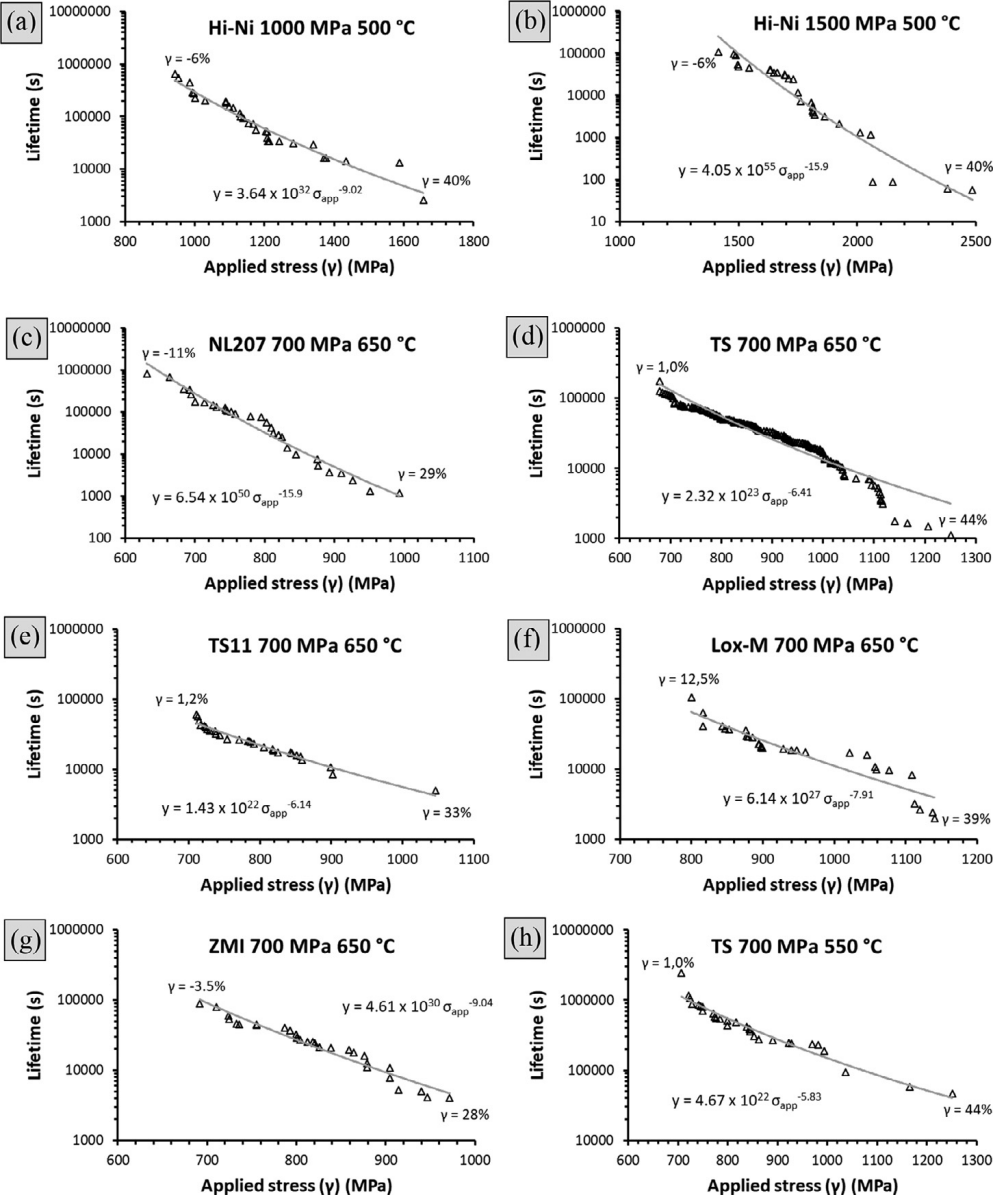
Endurance diagram built combining lifetime and γ-induced strength scattering for (a,b). Hi-Ni at 500 °C and 1000 or 1500 MPa, (c). NL207 (d). TS (e). TS11 (f). Lox-M and (g). ZMI at 650 °C and *σ_app.t(γ=0%)_* = 700 MPa (h). TS at 550 °C and 700 MPa.

Above theories neglect a key factor: the uncertainty on the stress applied to the tow probe, affecting the crack growth kinetic. The section of a tow is indeed strongly method related and varies from probe to probe [2], with a stress misestimation up to several hundreds of MPa [1,2]. As a first step, this concept was used to build endurance diagrams as shown in Fig. 5, from *t*_f.t_ and γ (Eq. (3)) datasets given in [2] and gather in the supplementary file ‘Distribution association.xlsx’. If the stress exponents estimated this way are globally overestimated as shown in Table 5, the approach comprise the elegancy not invoking other variability sources (stick to the unbiased bundle model). On TS11 and ZMI type, values were close to the expected ones (respectively 6.1 and 9 against 5.8 and 9.1). Largest discrepancies were found for Hi-Ni under *σ_app.__t_* = 1500 MPa (*n_est_* = 16 against 8.4) and NL207 under *σ_app.__t_* = 700 MPa (*n_est_* = 16 against 7.2). In these conditions, because some tows were failing during the loading step (strength close to the applied stress, Table 4) and consequently discarded, the *t*_f.t_ dispersion is most likely biased. Moreover, it is worth reminding these statistical parameters were extracted from a limited dataset size (commonly 30 values) and thus do not depict its full range.

**Table 5:** Comparison of stress exponents extracted from experimental datasets (*n_true_*) or from the association of lifetime and γ respective variabilities (*n_est_*) (Fig. 5).

Condition	n_true_	n_est_
Hi-Ni 1000 MPa 500 °C	8.4	9.0
Hi-Ni 1500 MPa 500 °C	8.4	16
NL207 700 MPa 650 °C	7.3	16
TS 700 MPa 650 °C	5.0	6.4
TS11 700 MPa 650 °C	5.8	6.1
Lox-M 700 MPa 650 °C	4.9	7.9
ZMI 700 MPa 650 °C	9.1	9.0
TS 700 MPa 550 °C	5.0	5.8

The approach was hence transcribed to the above algorithm. Each tow was given a structure, summarized by its effective section fraction (*S_t_* x (1-*γ*), Eq. (3)), and each of the N_0_ filaments was given a strength, randomly selected among the 2-parameters Weibull distribution (Table 1) [1]. From this set of data, the virtual time to failure for each filament (Eq. (13)) was calculated and ranked. A weakest link approach finally gave the bundle behavior. Table 6, Table 7, Table 8 show an example of this algorithm applied to Hi-Ni-S tested at 600 °C and *σ_app.t(γ__=__0%)_* = 300, 600 and 900 MPa. For 20 random tows, these tables gather the strength of the critical filament, narrowly dispersed as awaited after Fig. 1, γ and the associated virtual time to failure. On this fiber type (*N_t_* = 500, $\bar{\gamma }$ = 25% and *α_t_* = 8.22%), the critical filament rank equals 30. It can be noticed critical filaments are systematically weak, strength probability *P_i(30_*_)_<0.15 and *σ_f.f_*<2400 MPa, whatever the virtual performance. This latter looks better related to γ covering 3 to 4 orders of magnitude when γ varies from 5 to 95%. Moreover, null virtual lifetime were observed for *P_i30_*>0.98 and 0.95 at respectively 600 and 900 MPa (when the applied stress exceeds *σ_f.f(αt)_*).

**Table 6:** Summary of 20 simulation runs for Hi-Ni-S tows under *σ_app.t(γ__=__0%)_* = 300 MPa and 600 °C. *Pi_(30)_* describes the strength probability for the 30th fiber to fail (α_t_ fraction) and *P_γ_* the γ probability of the tow.

t_f.t_ (s)	P_i(30)_	σ_f.f(αt)_ (MPa	P_γ_	γ
3.00 × 10^9^	0.095	2330	0.062	0.040
1.45 × 10^9^	0.063	2215	0.211	0.100
1.00 × 10^9^	0.064	2216	0.331	0.145
3.83 × 10^7^	0.090	2313	0.887	0.473
2.25 × 10^9^	0.078	2273	0.110	0.061
7.05 × 10^8^	0.078	2272	0.473	0.200
5.32 × 10^8^	0.087	2302	0.559	0.238
2.02 × 10^9^	0.090	2312	0.174	0.086
6.88 × 10^8^	0.063	2214	0.442	0.188
2.56 × 10^8^	0.107	2363	0.720	0.325
1.54 × 10^8^	0.100	2345	0.782	0.368
8.07 × 10^8^	0.072	2252	0.260	0.180
4.90 × 10^8^	0.079	2276	0.565	0.241
2.18 × 10^8^	0.081	2285	0.718	0.323
2.39 × 10^9^	0.083	2290	0.103	0.058
2.84 × 10^8^	0.091	2318	0.689	0.305
6.44 × 10^5^	0.107	2363	0.979	0.705
5.27 × 10^8^	0.096	2332	0.576	0.246
1.37 × 10^9^	0.057	2188	0.209	0.099
3.04 × 10^9^	0.090	2312	0.047	0.033

**Table 7:** Summary of 20 simulation runs for Hi-Ni-S tows under *σ_app.t(γ__=__0%)_* = 600 MPa and 600 °C. *P_i(30)_* describes the strength probability for the 30th fiber to fail (α_t_ fraction) and *P_γ_* the γ probability of the tow.

t_f.t_ (s)	P_i(30)_	σ_f.f(αt)_ (MPa	P_γ_	γ
6.66 × 10^5^	0.086	2301	0.816	0.397
1.98 × 10^6^	0.093	2324	0.686	0.304
8.57 × 10^6^	0.100	2344	0.351	0.152
8.18 × 10^6^	0.056	2183	0.247	0.113
1.19 × 10^7^	0.092	2320	0.228	0.106
1.84 × 10^6^	0.080	2280	0.681	0.301
1.53 × 10^7^	0.054	2171	0.040	0.029
7.97 × 10^4^	0.051	2159	0.921	0.526
1.46 × 10^7^	0.083	2289	0.137	0.072
8.63 × 10^5^	0.085	2298	0.790	0.374
1.55 × 10^6^	0.090	2312	0.720	0.324
7.53 × 10^6^	0.073	2252	0.327	0.143
6.28 × 10^5^	0.088	2308	0.858	0.403
1.48 × 10^7^	0.050	2151	0.036	0.027
1.83 × 10^4^	0.074	2256	0.960	0.620
1.86 × 10^7^	0.128	2419	0.151	0.077
0	0.074	2259	0.985	0.747
2.33 × 10^6^	0.077	2269	0.635	0.275
4.25 × 10^6^	0.090	2315	0.527	0.224
2.67 × 10^7^	0.097	2335	0.003	0.005

**Table 8:** Summary of 20 simulation runs for Hi-Ni-S tows under *σ_app.t(γ__=__0%_*_)_ = 600 MPa and 600 °C. *P_i(30)_* describes the strength probability for the 30th fiber to fail (α_t_ fraction) and *P_γ_* the γ probability of the tow.

t_f.t_ (s)	P_i(30)_	σ_f.f(αt)_ (MPa	P_γ_	γ
1.92 × 10^5^	0.074	2258	0.532	0.226
1.42 × 10^4^	0.076	2266	0.868	0.704
1.34 × 10^5^	0.085	2296	0.628	0.272
3.72 × 10^5^	0.110	2372	0.429	0.183
8.38 × 10^2^	0.080	2279	0.944	0.576
9.52 × 10^5^	0.107	2363	0.126	0.067
5.03 × 10^4^	0.062	2208	0.746	0.342
4.98 × 10^3^	0.067	2231	0.908	0.504
6.36 × 10^4^	0.092	2320	0.752	0.346
2.70 × 10^5^	0.101	2346	0.499	0.212
1.82 × 10^5^	0.096	2331	0.582	0.249
2.59 × 10^5^	0.098	2338	0.178	0.214
0	0.097	2334	0.964	0.635
2.72 × 10^4^	0.101	2346	0.840	0.419
9.36 × 10^5^	0.079	2276	0.071	0.044
2.54 × 10^5^	0.087	2302	0.488	0.207
1.32 × 10^5^	0.087	2303	0.634	0.275
3.59 × 10^5^	0.097	2334	0.415	0.177
8.70 × 10^4^	0.075	2263	0.689	0.305
4.11 × 10^4^	0.068	2233	0.777	0.364

**Fig. 6:**
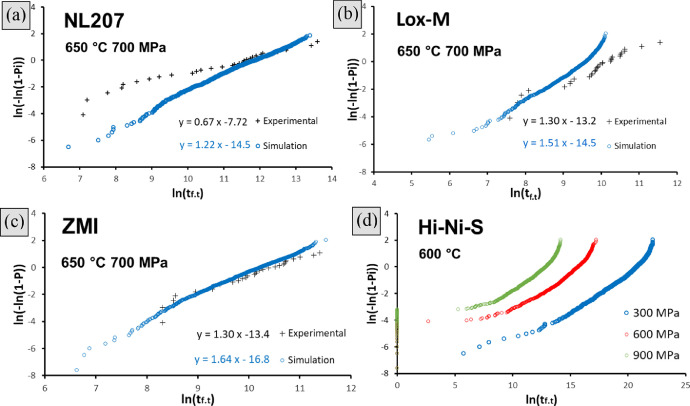
Weibull plot of experimental and Monte Carlo simulated times to failure including the dispersion on effective bundle section (γ) (Eq. (11)) for (a). NL207 (b). Lox-M, (c). ZMI at 650 °C under *σ_app.t(γ=0%)_* = 700 MPa or (d). Hi-Ni-S at 600 °C and 300, 600 or 900 MPa.

**Fig. 7:**
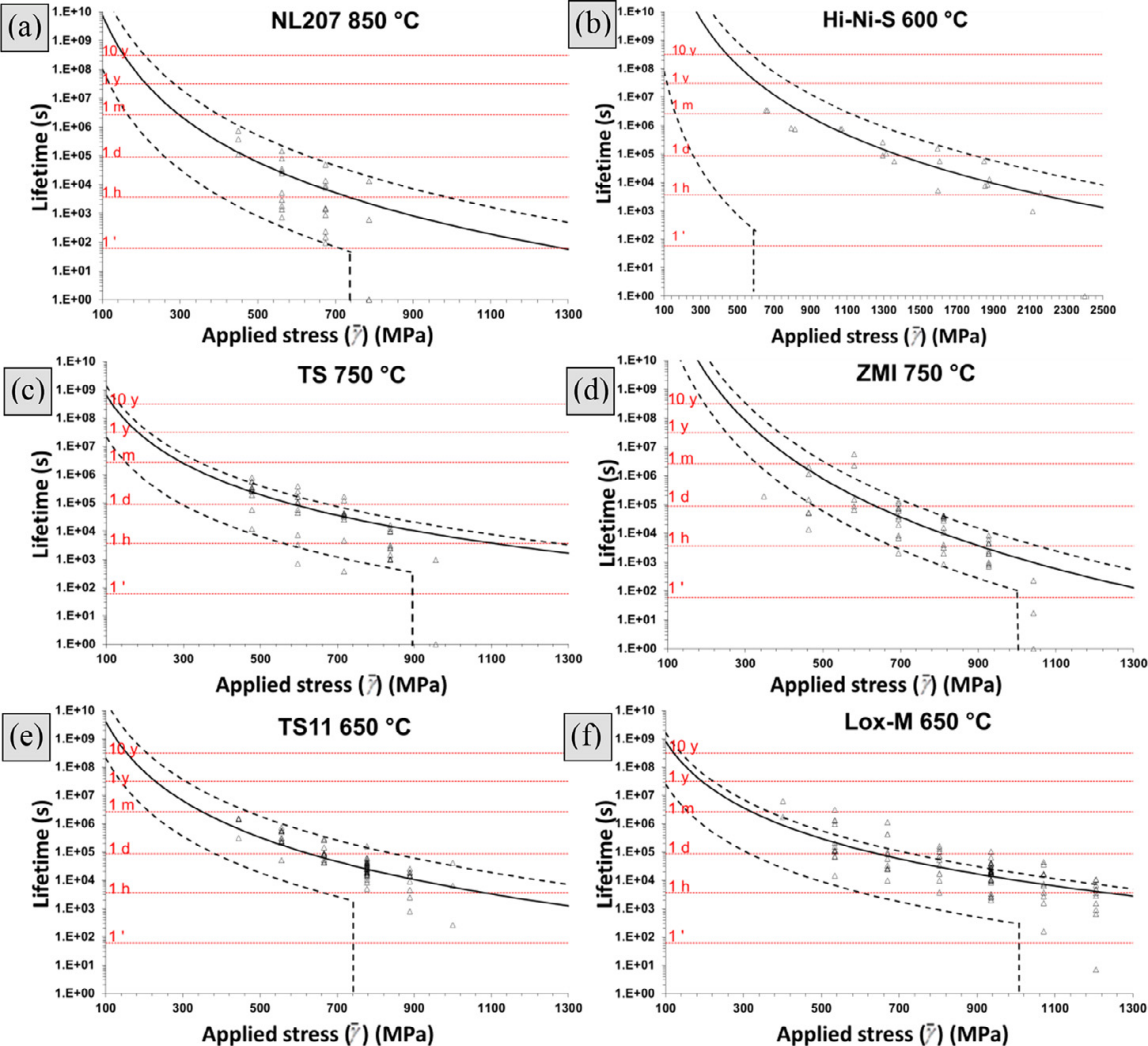
Stress-time to failure diagrams for (a). NL207 at 850 °C, (b). Hi-Ni-S at 600 °C, (c). TS or (d). ZMI at 750 °C, (e). TS11 or (f). Lox-M at 650 °C comparing experimental and Monte Carlo simulated range. Variation on tow stress was introduced into the simulation, A_1_ coefficient represented the median lifetime.

The tremendous impact stress misestimation plays on time to failure dispersion is illustrated on Weibull diagrams (Fig. 6). On Lox-M and ZMI types, the comparison of Fig. 2d,e with Fig. 6b,c highlights the increase of virtual lifetime range obtained, almost encompassing the experimental results. Extended to different applied stresses ranging from 100 to 1400 MPa, endurance diagrams were constructed displaying the median time to failure as well as its simulated range, then compared with experimental data points (Fig. 7). This visualization helps to identify the stress for which loading failure would be expected (600 MPa on above Hi-Ni-S mentioned fiber type and above 700 MPa on the other types). With this approach NL207, Hi-Ni-S, TS and ZMI results are consistent to each other (no discrepancy in simulated range) unlike above approaches. A slight underestimation of the predicted scattering compared to the experienced one is to be noticed on all types. On Lox-M tows however, this computation clearly underestimate experimental values. Table 9 gathers the coefficients *A* for the median, the upper and the lower virtual lifetimes as extracted from simulations.

**Table 9:** Prediction parameters used as simulation input (*A_1_*) or describing the simulation output (median, minimal and maximal lifetime).

	*A*_1_	*A*	*A*_min_	*A*_max_
NL207 850 °C	1.54 × 10^8^	3.0 × 10^24^	4.0 × 10^22^	2.6 × 10^25^
Hi-Ni-S 600 °C	1.41 × 10^12^	3.2 × 10^27^	2.0 × 10^22^	2.2 × 10^28^
TS 750 °C	1.31 × 10^9^	6.0 × 10^18^	2.1 × 10^17^	1.5 × 10^19^
ZMI 750 °C	2.24 × 10^8^	3.0 × 10^30^	2.4 × 10^29^	1.1 × 10^31^
TS11 650 °C	2.36 × 10^10^	1.9 × 10^21^	8.3 × 10^19^	9.4 × 10^21^
Lox-M 650 °C	4.46 × 10^10^	5.0 × 10^18^	1.5 × 10^17^	1.3 × 10^19^

## 2 Experimental Design, Materials and Methods

### 2.1 Material

Polymer derived SiC-based fibers presented in this dataset were provided by Nippon Carbon Co. Ltd. or UBE Industries Ltd. Different processing routes, leading to different generations, were tested: the first oxygen cured generation (Nicalon® NL207, Tyranno® Grade S, referred as TS, Tyranno® Lox-M, and Tyranno® ZMI), the second electron-beam cured generation (Hi-Nicalon® named Hi-Ni) and the third generation which underwent a high temperature annealing treatment (Hi-Nicalon® Type S named Hi-Ni-S) [4,5]. Two different TS fiber diameters were studied (8.5 µm or 11 µm, the later named TS11). Their respective properties were given in [1].

### 2.2 Method

The same bundle probes were used for tensile or static fatigue testing. Sized bundles of 300 mm length (*L_0_*) were weighted (*m*_0_, Eq. (2)) and positioned in alumina tube grips. To ensure probe alignment, a pre-load was applied and maintained by fugitive Loctite® glue. A solution of dissolved PMMA was applied on the 25 mm gage length separating the grips, to avoid capillarity cement transportation during curing. Tubes were finally filled with alumina based thermostructural cement (Ceramabond 503, Polytec PI) and cured at 370 °C for 2 h. The engineering stress applied to the bundle, corrected accounting for the fraction of unloaded fibers (*γ* Eq. (3)), inferred from Eq. (1).

(1) $$\sigma_{\gamma }=\frac{w_{t}}{S_{t}\left(1-\gamma \right)}$$

with

(2) $$S_{t}=\frac{m_{0}}{L_{0}\rho }$$

and

(3) $$\gamma =1-\frac{N_{0}}{N_{t}}=1-\frac{E_{t}}{E_{f}}$$

Where *ρ* is the fibers density, *w_t_* the applied force, *N_0_* the initial number of intact filaments and *N_t_* its total manufactured number (500, 800 or 1600). *E_t_* and *E_f_* are respectively monofilament and tow Young's moduli.

The effective tow section (S_t_ (1-*γ*)) differs from probe to probe. Its dispersion was satisfactorily fitted by a Weibull statistical distribution law (Eq. (4)) as shown in [2], where *m*_γ_ is the modulus, *γ_0_* the characteristic fraction and *P_γ_* the probability.

(4) $$\gamma =\gamma_{0}\ln{\left(\frac{1}{1-P_{\gamma }}\right)}^{\frac{1}{m_{\gamma }}}$$

Tensile tests were carried out at a constant displacement rate of 50 µm min^−1^. Under these conditions (no load sharing), the bundle model considers filaments break progressively and individually as the force applied to them reach a critical value [[6], [7], [8]]. The maximal force is met when the ratio (*α*) of broken filaments (*N*) to the large total number (*N_0_*) reaches a critical ratio (*α_c_*) assumed from filament strength distribution (Weibull statistic with *m*_f_ as modulus and *σ_1.f_* as characteristic strength) as follows [9], [10], [11] (Table 2):

(5) $$\alpha_{c}=\frac{N_{c}}{N_{0}}=1-e^{\left(\frac{-1}{m_{f}}\right)}$$

Static fatigue experiments were however conducted in a vertical resistive furnace opened to atmospheric environment suspending a dead weight (applying a constant force *w*_t_) to the lower grip and initiating the heating up. Only probes that survived to loading step were considered. The automatic stop of timer when specimen failed gave the tow lifetime. The experimental setup was shown in [1] and [3]. Because force and strain could not be recorded, the actual γ value of the tested tow could not be estimated (*E_t_* unknown). Its average value, extracted from tensile tests [2], was hence used. Tests were performed at different stresses (11 tests per condition) to construct endurance diagrams. Some conditions were more extensively tested for scattering assessment purpose. Lifetime variability can nicely be described using the Weibull statistic (Eq. (6)) [14,15], where t_f.t0_ is the characteristic time to failure and *m_df.t_* the static fatigue Weibull modulus. All these statistical parameters were assessed by linear least square method applied to Weibull plots.

(6) $$P_{i}=1-e^{-{\left(\frac{t_{f.t}}{t_{f.t0}}\right)}^{m_{df.t}}}$$

As the stress is increased, a lower lifetime is expected due to accelerated SCG mechanism. This lifetime evolution can properly be predicted from the power law relating the crack velocity to the stress intensity factor (*K_I_*):

(7) $$v=\frac{da}{dt}=V*{\left(\frac{K_{I}}{K_{IC}}\right)}^{n}$$

Where K_I_ is inferred from the fracture mechanics law :

(8) $$K_{I}=\sigma_{app}Y\sqrt{a}$$

*Y* is the flaw shape factor (2 π^−1/2^ for a penny shaped crack), *v* is the crack velocity, *a* its length, *t* the time, *K_IC_* the critical stress intensity factor *n* and V* respectively material and environmental constants. V* K_IC_^−^^n^ was substituted by A_1_ below. The time to failure for a given filament under constant stress is caused by the growth of a flaw from its initial (*a_i_* (9)) to critical (*a_c_* (10)) size:

(9) $$a_{i}=K_{IC}^{2}/\sigma_{f.f}^{2}Y^{2}$$

(10) $$a_{c}=K_{IC}^{2}/\sigma_{app.f}^{2}Y^{2}$$

This statement is solved substituting Eq. (8) into Eq. (7) and integrating it:

(11) $$t=\int_{a_{i}}^{a_{c}}\frac{da}{v}=\int_{a_{i}}^{a_{c}}\frac{da}{A_{1}K_{I}^{n}}=\frac{1}{A_{1}\sigma^{n}Y^{n}}\int_{a_{i}}^{a_{c}}a^{-\frac{n}{2}}da$$

Integration gives the following:

(12) $$t_{f.f}=\frac{1}{A_{1}\sigma_{app}^{n}Y^{n}}\frac{2}{n-2}\left[a_{i}^{\frac{2-n}{2}}-a_{c}^{\frac{2-n}{2}}\right]$$

Inserting the relations (9) and (10) into Eq. (12) yields the following equation for the filament time to failure:

(13) $$t_{f.f}=\frac{K_{IC}^{2-n}}{A_{1}\sigma_{app.f}^{n}Y^{2}}\frac{2}{n-2}\left[\sigma_{f.f}^{n-2}-\sigma_{app.f}^{n-2}\right]$$

This expression can be simplified to the commonly assumed power law when *σ_app_<<σ_f.f_*.

(14) $$t_{f.f}=A\sigma_{app}^{-n}$$

With:

(15) $$A=\frac{K_{IC}^{2-n}}{A_{1}Y^{2}}\frac{2}{n-2}$$

The above formulation describe the time to failure for a single filament. Under constant force conditions, the delayed failure of a multifilament tow (*t_f.t_*) occurs once a critical amount of fibers, breaking individually and sharing their load homogeneously, had failed [12]. This fraction stems from Eq. (16), as described in [13], with a tensile strength deduced from filament Weibull distribution (Eq. (17)). Stress exponent are given in Table 5 and Weibull moduli for filaments in the companion manuscript.

(16) $$\alpha_{t}=1-e^{\left(-\frac{n-2}{nm_{f}}\right)}$$

(17) $$\sigma_{f.f}=\sigma_{1.f}\ln{\left(\frac{1}{1-P_{i}}\right)}^{\frac{1}{m_{f}}}$$

Inserting the relation (17) into Eq. (13) yield the following expression for *t_f.t_* (tow time to failure):

(18) $$t_{f.t}=\frac{K_{IC}^{2-n}}{A_{1}Y^{2}\sigma_{app.t}^{n}}\frac{2}{n-2}\left[{\left[\sigma_{{}_{1.f}}^{n-2}\ln\left(\frac{1}{1-\alpha_{t}}\right)\right]}^{\frac{n-2}{m_{f}}}-\sigma_{app.t}^{n-2}\right]$$

This formulation can further be adjusted integrating the variability on the applied stress to tow. Eq. (19) thus arise integrating relations (1) and (4) in place of *σ_app.f(αt)_* in Eq. (18). The Monte Carlo algorithm used to construct Figs. 6 and 7 considered a random probability *P_γ_* (ranging from 0 to 1) for each tow.

(19) $$\begin{array}{ccc} t_{f.t} & = & \frac{K_{IC}^{2-n}}{A_{1}Y^{2}{\left(\frac{w_{t}}{S_{t}(1-\gamma_{0}\ln{\left(\frac{1}{1-P_{\gamma }}\right)}^{\frac{1}{m_{\gamma }}})}\right)}^{n}}\\ & & \times \;\frac{2}{n-2}\left[\sigma_{{}_{1.f}}^{n-2}{\left[\ln\left(\frac{1}{1-\alpha_{t}}\right)\right]}^{\frac{n-2}{m_{f}}}-{\left(\frac{w_{t}}{S_{t}(1-\gamma_{0}\ln{\left(\frac{1}{1-P_{\gamma }}\right)}^{\frac{1}{m_{\gamma }}})}\right)}^{n-2}\right] \end{array}$$

## CRediT Author Statement

**S. Mazerat**: Conceptualization, Methodology, Software, Formal analysis, Investigation, Data Curation, Writing - Original Draft, Writing - Review & Editing, Visualization; **R. Pailler**: Validation, Resources, Supervision, Project administration, Funding acquisition.

## Declaration of Competing Interest

The authors declare that they have no known competing financial interests or personal relationships which have, or could be perceived to have, influenced the work reported in this article.
